# The initial validation of an **E**vidence-informed, compete**n**cy-based, **A**pplied **C**ompassion **T**raining (EnACT) program: a multimethod study

**DOI:** 10.1186/s12909-024-05663-0

**Published:** 2024-06-21

**Authors:** Shane Sinclair, Swati Dhingra, Shelley Raffin Bouchal, Cara MacInnis, Daranne Harris, Amanda Roze des Ordons, Barbara Pesut

**Affiliations:** 1 Faculty of Nursing, University of Calgary, Calgary, Canada; 2 Cumming School of Medicine, University of Calgary, Calgary, Canada; 3 Compassion Research Lab, University of Calgary, Calgary, Canada; 4 Department of Psychology, Acadia University, Wolfville, Canada; 5 School of Nursing, University of British Columbia, Kelowna, Canada

**Keywords:** Compassion, Compassionate care, Training, Education, Competence

## Abstract

**Introduction:**

Compassion is positively associated with improved patient outcomes, quality care ratings, and healthcare provider wellbeing. Supporting and cultivating healthcare providers’ compassion through robust and meaningful educational initiatives has been impeded by a lack of conceptual clarity, inadequate content coverage across the domains of compassion, and the lack of validated evaluation tools. The EnACT program aims to address these gaps through an **E**vidence-informed, compete**n**cy-based, **A**pplied, **C**ompassion **T**raining program delivered to healthcare providers working in various clinical settings. In this study, we describe the development and initial validation of the program, which will inform and be further evaluated in a forthcoming Randomised Controlled feasibility Trial (RCfT).

**Method:**

A multimethod design was used to explore learner needs, experiences, and outcomes associated with the program. Pre- and post-training surveys and qualitative interviews (1 month post training) were conducted among twenty-six healthcare provider learners working in acute care and hospice. Quantitative measures assessed professional fulfillment/burnout, self-confidence in providing compassion, learner satisfaction, and compassion competence. Qualitative interviews explored learners’ experiences of the program, integration of learnings into their professional practice, and program recommendations.

**Results:**

Learners exhibited relatively high self-assessed compassion competence and professional fulfillment pre-training and low levels of burnout. Post-training, learners demonstrated high levels of compassion confidence and satisfaction with the training program. Despite high levels of reported compassion competence pre-training, a statistically significant increase in post-training compassion competence was noted. Thematic analysis identified five key themes associated with learners’ overall experience of the training day and integration of the learnings and resources into their professional practice: (1) A beginner’s mind: Learner baseline attitudes and assumptions about the necessity and feasibility of compassion training; (2) Learners’ experiences of the training program; (3) Learner outcomes: integrating theory into practice; (4) Creating cultures of compassion; and (5) Learner feedback.

**Conclusion:**

Findings suggest that the EnACT program is a feasible, rigorous, and effective training program for enhancing healthcare provider compassion. Its evidence-based, patient-informed, clinically relevant content; interactive in class exercises; learner toolkit; along with its contextualized approach aimed at improving the clinical culture learners practice holds promise for sustaining learnings and clinical impact over time—which will be further evaluated in a Randomized Controlled feasibility Trial (RCfT).

## Introduction

While conceptualizations of compassion vary within the broader literature [1–4], within healthcare a series of systematic reviews [5–9] and empirical studies [10–19] delineating the core components of the construct, defined compassion as *“a virtuous response that seeks to address the suffering and needs of a person through relational understanding and action”* [10 p195]. The significance of compassion in enhancing health outcomes and quality care ratings of patients [20–22] and healthcare providers’ (HCPs) workplace well-being has been broadly reported in the healthcare literature [5, 6, 23]. Providing compassionate care has been reported to increase HCP job satisfaction and retention, while also reducing moral distress, burnout, and occupational stress [24, 25]. While there is emerging evidence that HCP compassion can be cultivated [4, 12–14], there still remains a lack of evidence-based clinically relevant training that focuses on both enhancing HCPs compassion competence and importantly, creating the organizational conditions for their compassion to flourish [7, 11, 26].

While patients, HCPs, and educators believe compassion can and should be integrated into training for both practicing and future HCPs [5, 12, 13, 27] recent systematic reviews and qualitative studies of international leaders in compassion education identified challenges [7, 11, 14, 28]. These include: a lack of conceptual clarity; inadequate content coverage across the domains of compassion; the need for a competency-based approach; insufficient integration of evidence informed and learner centric teaching methods; the lack of valid measures to evaluate retention, competence, clinical impact, and patient reported outcomes; and finally, the need to engage organizational leaders to address system factors impacting learners’ ability to provide compassion in their clinical practice [7, 11, 14, 24, 29–32].

Having recently developed a model of compassion across various clinical settings [10, 15–18, 33] and developed and validated a patient reported compassion measure [34, 35], we aimed to develop a compassion training program for HCPs to address the aforementioned gaps.

### Study aims and objectives

The overarching purpose of the EnACT program is to provide HCPs and leaders with a feasible, empirically grounded, competency-based, applied, multimodal, training program that assesses learner and patient outcomes over time with valid and reliable measures. The aim of this study was to: (1) describe how the training program was developed and; (2) report on the results from the implementation of the initial phase of the training program among a group of HCP learners.

## Research questions

What are learners’ needs, experiences, and outcomes associated with the pilot phase of the EnACT program?

### Development of the EnACT curriculum

**Fig. 1:**
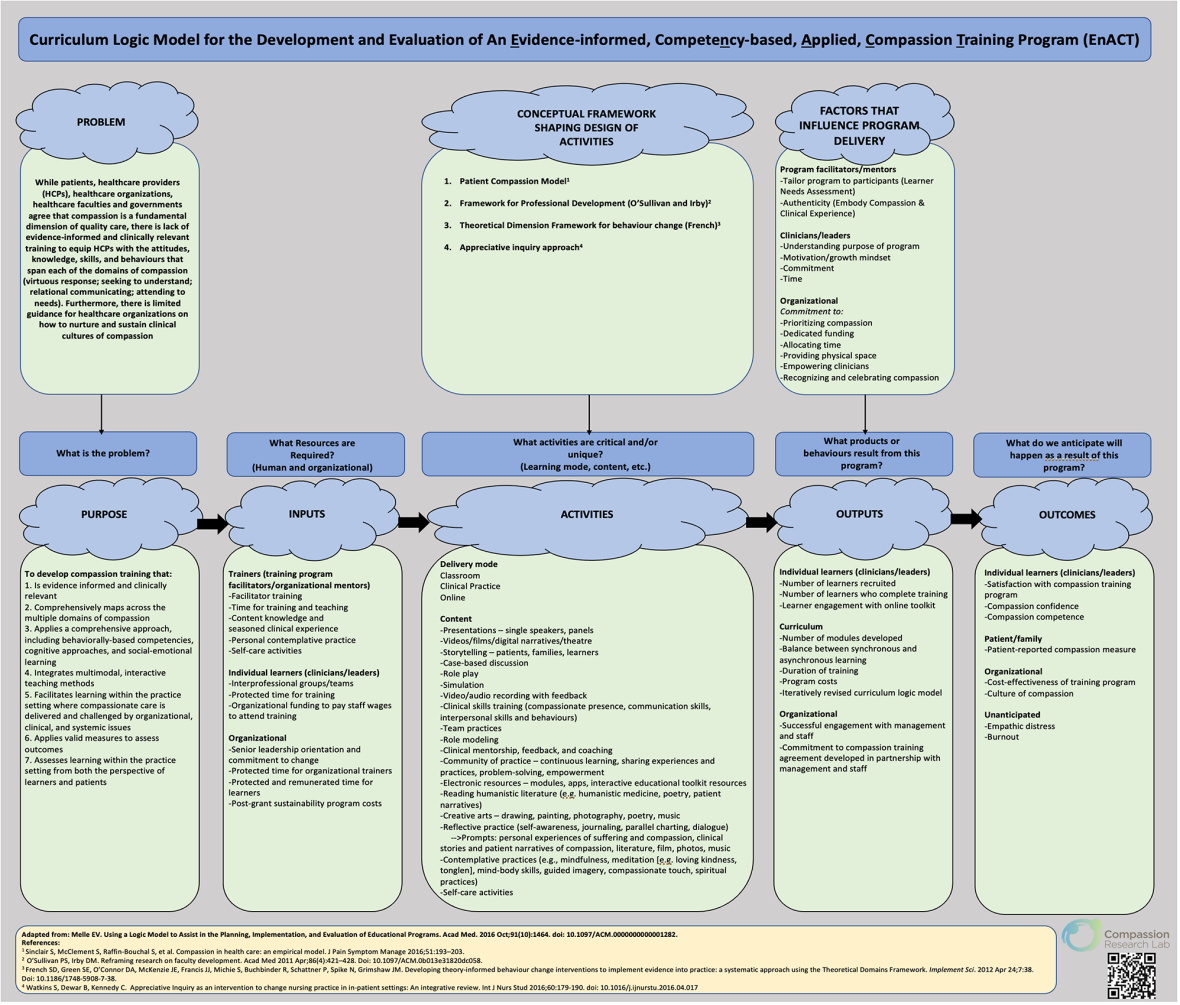
Curriculum Logic Model for the Development and Evaluation of EnACT

The EnACT curriculum was generated out of our programmatic research on compassion across a variety of patient and HCP populations [10, 6, 15–18, 34]. A curriculum logic model (Fig. 1) was first developed [36], in consultation with both the clinical training sites and a Patient and Family Advisory Council (PFAC), to guide this process. Over the course of a year, the pilot curriculum (content, methods, activities, and learner resources) was developed by members of the research team, consisting of subject matter and curriculum design experts, resulting in a one-day (6.5 h) training program intended for HCPs and leaders. The program is comprised of four modules, that mirror the domains of the compassion model [10] —1) Virtuous Response; 2) Seeking to Understand; 3) Relational Communicating; and 4) Attending to Needs. The one-day program was first delivered to members of the PFAC and a group of undergraduate and graduate student nurses, with feedback being integrated into the program in an iterative manner.

### Learner needs assessment

In keeping with best practice guidelines [37, 38], prior to piloting the one-day EnACT curriculum among a group of HCPs within both acute care and hospice settings, we conducted a learner needs assessment (LNA) at each of the participating sites to assess learner needs and to identify personal, relational, and systematic barriers in integrating compassion within their clinical setting. The LNA was administered to staff (*n* = 55) via REDCAP survey software, with barriers to compassion assessed with Likert scales and open-ended questions (see Table 1). Compassion competence was assessed using the Sinclair Compassion Questionnaire-Health Care Provider Competency Self-Assessment (SCQ-HCPCSA) [24]. The SCQ-HCPCSA covers five domains of compassion which include: virtuous response, relational space, seeking to understand, relational communicating, and attending to needs [15, 34]. Survey and open-ended responses indicated that systemic barriers were perceived as greater impediments to compassion than personal or relational barriers, with the three main barriers identified being: (1) Healthcare system/organizational resistance and lack of support toward improving compassion; (2) Healthcare systems that are overly focused on biomedical, task-based, and economic outcomes and; (3) Issues related to professional burnout. Likewise, HCP respondents’ open-ended responses identified a number of recommendations for improving compassion in their workplace: (1) Management and organizational leadership involvement in improving compassion; (2) Greater availability of self-care resources; and (3) Ongoing education on providing compassion within a healthcare context.

**Table 1:** Compassion learners needs assessment

Research has identified a number of challenges to compassion. Please indicate the EXTENT to which the following factors hinder your ability to show compassion towards patients: 1 – Never 2 – Rarely 3 – Sometimes 4 – Often 5 – Always					
Perceived Compassion Barrier items (*abbreviated)	***N***	***Max***	***Min***	***Mean***	***Std. Deviation***
**Personal Barriers**					
Personal frustrations*	55	1	5	2.29	0.809
Not knowing how to show*	55	1	4	1.98	0.913
Wanting to “fix” problems*	55	1	5	2.55	1.051
Compassion not priority*	55	1	4	1.42	0.686
Challenges in life*	55	1	4	2.25	0.751
**Workplace/systems Barriers**					
Time*	54	1	5	3.48	0.885
Professional demands*	54	1	5	3.30	0.983
Biomedical focus*	54	1	5	2.74	0.994
Task-oriented*	54	1	5	3.06	1.071
Inflexible work*	53	1	5	2.62	1.113
Healthcare system resistance*	54	1	5	3.07	0.929
Healthcare system outcomes*	53	1	5	3.13	1.127
**Relational Barriers**					
Frustration patients*	54	1	4	2.30	0.662
Biases towards groups	54	1	3	1.83	0.694
Frustration frequent hospitalization*	54	1	4	1.91	0.734
Patients not receptive*	54	1	4	2.52	0.693
***Open-ended Questions*** 1. What other barriers do you feel have impacted your ability to provide compassion in your practice? [Please explain] 2. What suggestions would you make for overcoming the barriers to compassion that you currently face? [Please explain] 3. How do you think your organization could help to address barriers to compassion? [Please explain]					

In response to this feedback, we modified the EnACT program by enhancing existing content focused on organizational compassion, burnout, and time constraints. Specifically, we: embedded additional self-reflection exercises in each module [7, 11, 14]; added content on the personal, relational and organizational challenges to compassion [11, 14, 26] and developed a weekly compassion journal with self-care exercises and additional resources aimed at sustaining and extending classroom learnings [7, 11, 14]. Finally, we integrated a compassion commitment exercise for HCPs and organizational leaders, asking them to make three commitments to enhance compassion in their practice and organization. After making these modifications, we piloted the EnACT program among HCPs working in both acute care and hospice in order to evaluate the program, learner experiences, and various learner outcomes.

## Methods

This study used a multimethod design, via a pre-post intervention survey and qualitative interviews. The study was approved by the Conjoint Health Research Ethics Board (#20–0907) at the University of Calgary.

### Participants and procedures

The 6.5-hour pilot training was delivered to a group of HCPs at their workplaces over the course of a single day. English-speaking HCPs (physicians, nurses, and allied health professionals) greater than 18 years of age, currently practicing in one of the participating clinical settings, were eligible to participate. After receiving notification of the training program via study posters shared on the units and through email, 26 HCPs signed up for the pilot training and provided informed written consent.

### Data collection

Survey data was collected at two time points during the training day, immediately prior to commencing the training program and immediately after the training program. Pre-training surveys included a participant demographic form, the Professional Fulfillment Index (PFI) and the SCQ-HCPCSA. The PFI is a 16-item validated survey (α = 0.86–0.92) comprised of two scales assessing HCPs professional fulfillment and burnout. Response options are measured on a five-point Likert scale (0 “*not at all true”* to 4 “*completely true”* for professional fulfillment items and 0 *“not at all*” to 4 *“extremely”* for burnout items) [39]. A mean of all items in each respective scale is computed, with higher scores in each corresponding scale indicating greater professional fulfillment and greater burnout. The SCQ-HCPCSA is a 15-item validated measure (α = 0.94) assessing the degree HCPs feel they have sufficient knowledge, judgment, and skill to provide compassion, with responses measured on a five-point Likert scale ranging from 1 (not at all competent) to 5 (completely competent) [24]. A mean of all items is computed, with higher scores indicating greater competence. The post-training surveys included a post-training satisfaction questionnaire created for the study, the SCQ-HCPCSA, and a confidence in compassion questionnaire created for the study, assessing an individual’s feeling of self-assurance arising from one’s appreciation for one’s compassionate abilities or qualities. The 15 items are measured on a five-point Likert scale ranging from 1 (not at all confident) to 5 (very confident), with a mean of all items being computed, with higher scores indicating greater confidence. Additionally, one month post training, each learner participated in a semi-structured qualitative interview (Table 2) focused on their experiences of the training day and integrating the program into their professional practice.

**Table 2:** Qualitative interview guide

1. Please tell us about your overall experience with the EnACT program?
2. In what ways did you find the program to be helpful? a. Was the program helpful in improving your knowledge about being more compassionate? Why or why not? b. What changes did you experience with respect to your compassionate care? Are there any particular skills that changed (communication, clinical behaviours, listening skills etc.)? c. What changes did you experience with respect to your attitudes about compassion?
3. What do you feel were the strengths of the program? a. We would appreciate your thoughts on the topics, content, format, presenters, and any other aspect of the program.
4. Do you feel there are any components of the program that should be modified or could be improved upon? If yes, what do you think could be changed? We would appreciate your ideas on topics, content, format, presenters, and any other aspect of the program. a. How might these components be improved?
5. What teaching methods (the way in which things were taught – experiential/reflective learning, exercises, presentation? ) were most effective for your learning? a. In which ways were they effective?
6. In thinking about future research aimed at creating compassionate clinical cultures and healthcare systems: What do you think needs to happen to create a compassionate health care system? How would you go about it? a. In which ways should this program be adapted to create compassionate clinical cultures and healthcare systems?
7. Is there anything that you would like to mention that we haven’t talked about in regard to the compassion training program? Any additional comments

### Data analysis

We analyzed the data consistent with a multimethod study design in which the quantitative and qualitative data were analyzed separately [40–42]. Quantitative analyses included computing scale averages, assessing descriptive statistics (means, standard deviations, frequencies), conducting a dependent samples t-test (to assess the pre-post difference in compassion competence) and a one sample t-test (to examine differences in competence between HCPs who participated in the LNA and HCP learners post-training). Analyses were performed using IBM SPSS version 29. Qualitative data was analyzed using thematic analysis [43], which entails a six-phase coding framework of familiarization of data; generation of codes; combining of codes into themes; reviewing themes; determining significance of themes; and reporting findings [44]. Three members (SS, SRB, SD) of the analysis team familiarized themselves with each transcript by first independently reading and generating initial codes (meaning units) recorded in the margins of each transcript. The analysis team met weekly to compare their respective codes and develop a coding schema which was utilized and modified through the same process for each transcript. The coding schema was refined in an iterative manner with each subsequent transcript, eventually generating a framework of themes and subthemes by examining coherent patterns in the coded data. To further maintain credibility and rigor through this process an audit trail was created, allowing other members of the research team to review and examine the analytical procedures and decision-making processes [45, 46].

## Results

A convenience sample of HCPs and support staff were included in this study, with the majority of participants being frontline HCPs. A total of 26 HCP participants completed the one-day training with 13 participants from acute care and 13 from hospice. On average, these learners had 12.35 years of experience in healthcare, with the majority being white (58%), and female (92%) (Table 3).

**Table 3:** Study participant demographics

Characteristics	*n*	%
Professional Affiliation		
Registered Nurse	16	62
Licenced Practical Nurse	4	15.2
Health Care Aide	2	7.6
Others	4	15.2
**Type of Employment**		
Full-time	9	34
Part-time	14	54
Casual	3	12
**Ethnicity**		
White	15	57.8
Asian	8	30.8
Mixed	2	7.6
Preferred not to disclose	1	3.8
**Religious Affiliation**		
No religious group indicated	9	34
Catholic	8	30.8
Protestant	5	20
Muslim	1	3.8
Buddhist	1	3.8
Others	2	7.6
**Gender**		
Female	24	92
Male	2	8

### Quantitative results

Learners reported low levels of burnout (*M =* 1.03; *SD =* 0.89) and high levels of professional fulfillment (*M = 2*.81; *SD = 0.77*) as measured by the PFI and relatively high levels of self-assessed compassion competence (*M* = 4.56, *SD* = 0.42) prior to training as measured with the SCQ-HCPCSA. Post training, learners indicated high compassion confidence (*M* = 4.70, *SD* = 0.48) and high satisfaction with the program (*M* = 4.88, *SD* = 0.30). Notably, there was a statistically significant difference in post (*M* = 4.75, *SD* = 0.32) versus pre-training compassion competence, *t* (24) = -2.84, *p* = .009. This corresponds to a Cohen’s *d* effect size of 0.30 which is in the small-to-medium range [47].

Compassion competence reported in the LNA was relatively high (*M* = 4.54, *SD* = 0.36). While not all LNA respondents (*n* = 55) participated in the pilot training program (*n* = 26), when comparing the mean of post-training compassion competence in the study to the mean of compassion competence in the LNA, a significant difference was also observed, *t* (25) = 3.22, *p* = .004, with no significant difference *t* (24) = 0.45, *p* = .655, in compassion competence being observed between the LNA and pre-training timepoints.

### Qualitative results

Five overarching themes (Table 4) emerged from the thematic analysis of the qualitative interviews describing HCP learners’ experience and feedback on the program: (1) A beginner’s mind: Learner baseline attitudes and assumptions about the necessity and feasibility of compassion training; (2) Learners’ experiences of the training program: curriculum, teaching methods, interactive exercises and the learning environment; (3) Learner outcomes: Integrating theory into practice; (4) Creating cultures of compassion: The need to create the conditions for compassion to flourish; and (5) Learner feedback: Suggested revisions and future considerations. Each qualitative theme is described below alongside participant exemplars that were selected based on representativeness and to reflect diverse opinions.

**Table 4:** Themes and subthemes

Themes	Subthemes	
**A beginner’s mind: Learner baseline attitudes and assumptions about the necessity and feasibility of compassion training**	The necessity of compassion training: A core but under-addressed and practice competency	
	The feasibility of compassion training: Learning what we thought we already knew	
**Learners’ experience of the training program: Curriculum, teaching methods, interactive exercises and the learning environment**	Curriculum	
	Teaching methods	
	The learning environment and interactive exercises	
	The overall learner experience	
**Learner outcomes: Integrating theory into practice**	Integrating training into personal and professional practice	Knowledge
		Attitudes
		Skills
	Sustaining Practices: Learner Toolkit	
**Creating cultures of compassion: The need to create the conditions for compassion to flourish**	Compassion champions: The role of senior leaders	
	Assessing patients’ experiences of compassion on a routine basis	
	Communities of compassionate practice: Establishing opportunities for ongoing learning and reflection as a team	
**Learner feedback: Suggested revisions and future considerations**		

### A beginner’s mind: Learner baseline attitudes and assumptions about the necessity and feasibility of compassion training

Learners identified a number of assumptions and attitudes that they brought with them into the classroom related to the topic of compassion and the notion of compassion training specifically.

### a) The necessity of compassion training: A core but under-addressed practice competency

There was broad consensus among learners that compassion is considered a core competency of HCPs and a reputed core value within their respective organizations. While its importance was unequivocally endorsed, some learners were initially skeptical about whether something so essential and seemingly simple needed to be formally taught. Upon taking the training however learners were unified in their belief that a compassion training program, when presented from an evidence and strength-based approach, was not only beneficial but a necessity that all HCPs should engage in on an ongoing manner.

*“It seems crazy that we as human beings need to be taught how to do this more effectively. But the insight into some very, I mean, what feels like it’s not rocket science, it’s simple things, but it is so important.” (Participant 4)*.

*“I think that like literally a course like this should be required by all clinical staff.” (Participant 1)*.

### b) The feasibility of compassion training: Learning what we thought we already knew

A second related sub-theme focused on the feasibility of compassion training, namely whether the topic of compassion could effectively be taught. Learners identified a number of pre-existing assumptions and attitudes that impacted their initial perceptions about the feasibility of compassion training including: perceiving compassion as a trait that some people possess and others do not; assuming that oneself is compassionate by virtue of being a HCP; and a tendency to overestimate one’s compassion competency. While learners felt that the compassion training program addressed these issues in a sensitive and evidence-based manner, they nonetheless acknowledged that these misperceptions could impact future recruitment, especially among HCPs who might not be aware of these assumptions.

*“I don’t want people to dismiss this course or dismiss the concept and the content because they think we’re already doing it. I don’t think we’re doing it as well as we think we are.” (Participant 2)*.

*“I guess I walked in thinking either you’re compassionate or you’re not.” (Participant 21).*

*“I mean, we all think we know what compassion is, turns out that we don’t know exactly everything we need to know about compassion. So, I think that was really helpful.” (Participant 12)*.

### Learners’ experience of the training program: Curriculum, teaching methods, interactive exercises, and the learning environment

Learners provided extensive feedback on their overall experience participating in the one day training program, including their insights on the content, teaching methods, and the more experiential elements of the program–the interactive exercises and learning environment.

*“At the end of a day everybody felt really enlightened and energetic about it.” (Participant 2)*.

*“It was one of those days that you just kind of kept in tune with the whole seminar.” (Participant 11)*.

“*I have really enjoyed and continue to enjoy the stimulation that’s came [from the training day], together with stuff that’s coming to my work. So it’s really good.” (Participant 4)*.

### a) Curriculum

Learners felt that the evidence-based content within the four modules, that mapped to the domains of an empirical model of compassion, was essential in legitimizing an important, but somewhat ephemeral topic, among a highly scientific, pragmatic, and critical audience of HCPs. Additionally, the fact that this empirical foundation was directly informed by patients and HCP accounts was perceived as being of equal importance, as it demonstrated the clinical relevance and utility of the content in the real world of clinical practice.

*“So, I think what I liked and what was helpful is hearing the background, like actual evidence-based information to line up with what we want to do at the bedside.” (Participant 1)*.

*“I think the content was really thoughtfully designed… the overarching background and a little bit of theory behind where these different aspects of compassion come from, what makes compassion different from empathy and sympathy, was also a highlight because they are different things, and it often gets generalized as one group.” (Participant 6)*.

*“I think that the facilitators were able to, you know, give real world examples and gave us space to listen to us as well.” (Participant 3)*.

### b) Teaching methods

Learners felt that the way in which the content was conveyed, through a variety of teaching methods, was imperative to the program’s success. The mixture of didactic, group discussions, self-reflection, case-based videos, and patient narratives was felt to not only keep learners engaged but honoured the diverse learning preferences and the multi-faceted ways that compassion can be expressed and experienced.

*“I also like that it’s broken into four modules. And within each module, there was some structure to it as far as an element of reflection, a little bit of didactic review or videos or sort of presentation components.” (Participant 2)*.

*“I liked the way that we were kind of guided through sort of our personal thoughts and then into the sort of professional research realm that has already been done and the feedback on the research that has been done and how we can actually follow up on that and make a difference on the ground.” (Participant 4)*.

*“I liked that there was not just one, one form for learning. Like I liked that, yes, [the facilitators] were standing at the front presenting, but sometimes there were videos that we watched and sometimes there was group discussion.” (Participant 15)*.

### c) The learning environment and interactive exercises

Learners highlighted the interactive exercises and having a learning environment where they learned alongside their colleagues, as important features of the compassion training program. Whether it was collectively establishing brave space learning principles at the outset, making individual compassion commitments at the completion of training, or engaging in a variety of applied skill-building exercises—these interactive elements brought the content to life in a personal and experiential manner, that learners felt would help them to sustain and apply the learnings outside of the classroom.

*“Looking back at my ‘circle of compassion’… in the training they had us talk about like if we could think of some patients that we’ve had recently who fell in our circle of compassion and then those that fell outside. So it was helpful to learn how to expand your compassion to include those harder patients.” (Participant 7)*.

*“So the times in which we had to do an activity like, hey, sit down with the person next to you and listen to how they have suffered and try to emotionally resonate with them. That probably felt uncomfortable for a lot of people, but I think it actually allowed you to experience invitational silence.” (Participant 1)*.

### d) The overall learner experience

While learners did provide some recommendations on how to further improve the program (see Learner Feedback section below), as a whole the program was enthusiastically received, including its delivery over the course of a single day.

*“I have nothing but good things to say about it. I actually thought it was really, really well done.” (Participant 2)*.

*“It was provided in a way that like usually these training programs go over multiple weeks, multiple days, like many different sessions. It was really nice to be able to just take the one day, have the full sit-down training session and just be able to focus on that.” (Participant 6)*.

*“Actually, the program for me, it’s perfect. I don’t see any improvement.” (Participant 18)*.

### Learner outcomes: Integrating theory into practice

In recognizing the programs focus on application in clinical practice, learners provided feedback on how they felt the knowledge, attitudes, and skills they developed were subsequently integrated into both their professional and personal lives.

### a) Integrating training into personal and professional practice

#### i. Knowledge

While each learner brought a baseline understanding of compassion to the training day, collectively they felt a strength of the program was clarifying, expanding, and challenging these pre-conceived notions from an evidence-based perspective. Specifically, learners appreciated learning about the clinical impact of compassion; individual differences in experiences and expressions of compassion; the role of self-care; demarcating compassion from sympathy and empathy; and the barriers and facilitators to compassion.

*“But I feel like there were things about being compassionate that I laughed at or didn’t know initially. So that was like a practical change for me.” (Participant 1)*.

*“Just gaining that insight as to what compassion really is and the different ways that it can be shown and I guess just brought across to our patients.” (Participant 9)*.

*“It was always viewed as like the golden standard–empathy. But then now with this compassion workshop, it’s like, well, the gold standard is actually compassion… It turns out I was like being empathetic, and that’s why I’m drained. So I think in a way, again, subtly, there’s like that little change in definition.” (Participant 5)*.

#### ii. Attitudes

In terms of attitude development, learners acknowledged having both their pre-existing attitudes challenged (e.g. cynicism toward compassion; values and biases affecting compassion to certain individuals, compassion being a ‘soft skill’, etc.) and cultivating new ones (e.g., self-awareness; intentionality; vulnerability, shared humanity) as a result of the training program.

*“I think it just really shows you where there’s opportunities to perhaps be even more intentional or where are the moments where you might not even realize that you’ve been holding your own biases and how that sort of shapes and shifts the way you end up treating others more compassionately.” (Participant 2)*.

*“I try to adjust my attitude now. Like if I just try to check in with myself, check in with my attitude, what my purpose is, I do try to be more intentional.” (Participant 7)*.

*“A lot of the things I kind of was already doing, but it just made me realize how important it really was… So it just gave me that awareness. And that’s something that I bring with me every day now.” (Participant 9)*.

#### iii. Skills

While most learners characterized their patient care as highly compassionate prior to the training, they nonetheless agreed that the program provided advanced skills that they applied to their subsequent practice. These included, but were not limited to, skills related to active listening; emotional resonance; non-verbal communication; and physical behaviours (e.g. sitting vs. standing, supportive touch, etc.).

*“I think I’ve been trying a lot more to just like use the silence and like make eye contact and that silence isn’t always offered that it’s okay to have silence in those moments. So, I think that was that was the big takeaway for me.” (Participant 12)*.

*“Just being more attentive to what other people say in that listening component as well. I try to listen more, because it is a skill.” (Participant 20)*.

*“Putting yourself in the patient’s shoes. It’s actually seeing them for what they’re going through beyond the bed number and the diagnosis. Who are they as a person? So that I feel changed for me.” (Participant 1)*.

*“I feel like I’m more purposeful and I notice more the person’s reaction to that. And I think, like personally, it’s made me definitely more mindful of how I do that and how to do it better.” (Participant 21)*.

### b) Sustaining practices: Learner toolkit

Learners were provided an opportunity to provide feedback on the development and components of a learner’s toolkit, that is intended to help sustain the learnings beyond the one-day training session. Potential components ranged from compassion checkpoint posters that could be displayed in the workplace, reflective workbooks, and clinical tip sheets.

*“I like the specific tools, like just the specifics around how you show compassion.” (Participant 21)*.

*“the [compassion] checkpoints and the posters and just kind of that cognitive reminder of doing that because it’s one of those things that will slide into the background. Otherwise, when you just kind of task focused in the work environment, but it’s a different thing.” (Participant 4)*.

### Creating cultures of compassion: The need to create the conditions for compassion to flourish

While equipping individual learners with the knowledge, attitudes, and skills to enhance compassion in their professional practice was felt to be essential, learners concurred that their work environments and their healthcare organizations played a critical role in enhancing compassion. In addition to existing curriculum and exercises within the training program focused on organizational compassion, learners shared their appreciation for how the program also sought to transform learners’ clinical settings.

*“So we need these personal skills for every individual health care provider, and then we need to help them be in environments where it’s easier to use those skills. Like it’s not enough to just train people to be more compassionate if you’re not giving them infrastructure environments where it’s easier to deliver that.” (Participant 16)*.

*“In order to, to achieve that compassionate care, we need, we keep asking nurses to be compassionate. But I think that we also need the healthcare system to be compassionate to nurses… like it’s just a circle.” (Participant 3)*.

### a) Compassion champions: The role of senior leaders

Learners underscored the value of engaging senior leaders in not only supporting the program, but in implementing the principles and educational resources into policy and practice. They felt that existing program features of having senior leaders make compassionate commitments to improve compassion in learners’ workplace, providing leaders with a summary of learner recommendations for improving compassion in the workplace, and eliciting leaders’ feedback on the organizational challenges they face in enacting compassion prior to program delivery were particularly beneficial components of the program. Additionally, learners felt it was imperative that clinical and administrative leaders, model compassion toward staff and actively champion compassion within their organization.

*“To have leaders that kind of support how important it is, I think would be another big piece. So having management and all that staff very involved. I think that would also probably help all our issues, the systemic issues if they learned that too.” (Participant 7)*.

*“Even your upper management [should receive training], like everybody because I think that would open up the door so that more people would be more compassionate and have a better understanding of compassionate care.” (Participant 11)*.

### b) Assessing patients’ experiences of compassion on a routine basis

In being informed about the proposed RCfT phase of the study learners provided their feedback on how they felt the impact of the training program could be further assessed. In addition to assessing learners’ satisfaction, compassion competence, professional fulfilment and burnout, participants felt it was essential to assess patients’ experiences of compassion pre and post training.

*“Some follow-up like actually measuring how our patients’ experience is, how do they feel compassion.” (Participant 1)*.

*“It would also be helpful to have, like not necessarily an audit, but again like an assessment of like where people were prior to the like implementation and then seeing what patient satisfaction scores are like following.” (Participant 6)*.

### c) Communities of compassionate practice: Establishing opportunities for ongoing learning and reflection as a team

Learners at each of the training sites shared how they had impromptu follow-up conversations within their clinical teams about aspects of the training and how it related to patient care. While these conversations flowed organically out of the course, learners felt that working with senior leaders to develop formal communities of practice, led by learners who were motivated to improve compassion within the workplace, was another tangible way to embed and sustain the principles and practices of the training program.

*“We did have a lot of conversations, like the co-workers that were at that compassion training, we had quite a few conversations in the following weeks about what we learned and how that kind of did translate into practice or like things would come up and be like well, ‘that is’ or ‘that isn’t very compassionate.’” (Participant 7)*.

*“Adding a reflective practice piece like how is this changing your practice? Just so we could have the discussion again with everyone like how’s it changed multiple people and then coming at it from this perspective.” (Participant 15)*.

### Learner feedback: Suggested revisions and future considerations

Although learners were asked how they felt the program could be improved (Table 2), the majority did not feel that any substantive revisions were needed. With further probing, a small number of learners suggested that enhancing the self-care components of the program and offering refresher courses were areas that could be further developed.

*“I don’t think anything really needs to be changed. Like, I honestly truly mean it when I was quite blown away by how well it was put together.” (Participant 1)*.

*“Like I adapted a lot of what you guys did provide for the self, like it applies. But it would be nice to hear an emphasis and like putting an importance on compassion to the self. I think that’s something that really lacks in healthcare. We’re always taught to like give and give and keep giving. It’d be nice to kind of have an instructor tell us like, ‘Hey, don’t forget about yourself. You deserve compassion too.’” (Participant 5)*.

*“I think it would be one of those programs you could take over again, because you probably learn something new each time you take it kind of thing. It would be neat to redo the whole like program as a follow-up piece just in a year or like six months.” (Participant 11)*.

## Discussion

While there are a number of educational interventions that utilize contemplative practices to enhance compassionate attitudes and feelings among HCPs, the EnACT program is unique as it: is rooted in a clinically relevant empirical model of compassion; is informed by systematic reviews and studies that revealed gaps in compassion training; is focussed on equipping learners with attitudes, knowledge, and skills that comprehensively map across the domains of compassion; and was developed by and for HCPs [7, 10, 11, 13, 14]. In doing so, the EnACT program addresses a number of the limitations of existing compassion training programs by incorporating robust evaluation measures, integrating clinically relevant scenarios and resources, including content and engaging organizational leaders to address system factors that inhibit learners’ ability to provide compassion, and focusing on the development of clinical skills that extend beyond contemplative practices aimed at enhancing self-awareness and compassionate feelings to others [11, 14, 24, 29, 31, 32].

Overall, learners were highly satisfied (*M =* 4.88) with the EnACT program—including those who expressed some initial reservation about the necessity and feasibility of compassion training. This positive learning experience seemed to be due to a combination of teaching strategies that have been identified as important components of continuing professional healthcare education in general and compassion training specifically. These include the integration of diverse teaching methods [11, 14, 48, 49], the use of learner needs assessments to contextualize the program to learner’s work environment [37, 38], the establishment of a positive and safe learning environment [50, 51], and the use of case-based clinical scenarios and self-reflection exercises to promote integration into practice [11, 49, 52–54]. While the quality of the curriculum and effectively evaluating its impact on learner and patient outcomes is essential, it seems that how the content is delivered is particularly salient when it comes to intrinsically personal and inherently relational topics such as compassion, where the requisite attitudes, knowledge, and skills are often ‘caught’ as much as they are ‘taught’.

A notable finding emerging from this study was a statistically significant increase in compassion competence, as measured by the SCQ-HCPCSA [24], from pre- to post-training. Similar results were reported by Pettit et al. [4] who found improvement in learners’ perception of personal compassion after participating in a compassion training program. In addition to providing promising initial evidence on the effectiveness of the EnACT training program, this finding stands in contrast to a recent study that reported a decline in HCP compassion after a training intervention [55]. This significant increase in compassion competence is particularly noteworthy in light of the fact that participants reported high levels of compassion competence prior to training (*M =* 4.56). Not only was the EnACT program able to produce a statistically significant improvement amongst a group of individuals who felt highly competent at baseline, recent research has demonstrated that even small differences in compassion, can have a significant and enduring impact on patients [6, 19]. While there may be concordance between self-assessed compassion competence and professional practice, this would require the addition of patient reports to ensure that HCPs perceived competence echo with patients’ actual experience—which will be assessed in the RCfT phase of this study using the SCQ [34].

The qualitative results of this study, suggest that high baseline self-reported compassion competence scores were influenced by a number of baseline attitudes and presumptions embedded within the sub-theme ‘The feasibility of compassion training: Learning what we thought we already knew’. While research suggests that most HCPs desire and perceive themselves to be innately compassionate [12, 13, 24], this does not necessarily translate to practice [56] or align with the perceptions of recipients and may, as suggested elsewhere [4], reflect an awakening in latent compassion that needs to be verified in actual practice. Specifically, individuals who are overly confident in their abilities are not only more likely to be ignorant of their deficiencies but also tend to over rate their abilities as a way to compensate for their lack of competency—a phenomenon known as the Dunning-Kruger effect [57–61]. This finding underscores the importance of creating a brave learning space to allow learners to safely explore these assumptions and the need for facilitators to apply a strength-based versus a deficiency-based approach to teaching and learning compassion (i.e. cultivating HCPs innate compassion versus teaching HCPs how to be compassionate) [50, 51, 62].

Results suggest that a vital, yet frequently overlooked component of compassion training programs, is the role that work environments play in facilitating, impeding, and sustaining HCPs ability to enact compassion outside the classroom. These results are consistent with other studies that have identified organizational and workplace issues as one of the most significant barriers to improving compassion in healthcare, leading some researchers to call for compassion to be considered as a key performance indicator within healthcare organizations and systems [4, 24, 26, 63–65]. While the principles of self-care, learner-centred education, self-reflection, and personal resilience are important concepts in both the classroom and personal practice, recent research demonstrates that they unfairly place the onus for systemic change, mitigating burnout, and improving compassion on individual HCPs [7, 11, 24, 66]. Likewise, our findings suggest that a compassion curriculum that focuses solely on the individual learner is insufficient, ineffective, and unsustainable. What seems to be essential, is acknowledging, addressing, and partnering with healthcare leaders prior to, during, and after training [67–69] —to facilitate system change, to transform the hidden curriculum (the unspoken values, beliefs, and norms of the practice culture) [70], to ensure that program learnings are sustained, and to create the conditions for HCPs compassion to flourish.

### Limitations

The findings from this study need to be interpreted within the context of some limitations. First, as this study assessed the initial validation of an emerging training program that will be further assessed through a RCfT, we had a relatively small sample of participants which impacted the results. Accordingly, while we identified a statistically significant change in compassion competency pre-post training, these findings need to be interpreted with some caution as we did not have sufficient statistical power to perform more complex analyses and we did not assess whether this effect was sustained over time. Second, given that enrollment in the training program was voluntary, we may have recruited HCP learners who were self-motivated to participate and excluded HCPs who were apathetic or critical to the topic. Third, although we attempted to recruit learners from diverse cultural backgrounds and disciplines, the sample predominantly consisted of White female registered nurse participants and the results may not be reflective of other cultures, genders, and disciplines. Finally, as this initial validation phase is one stage of a larger validation study, both the quantitative and qualitative results reported herein represent one stage in the validation of the training program, which need to be confirmed and further assessed amongst additional learners in the RCfT phase of the study.

### Implications

The results of this study will inform the RCfT phase of the EnACT study, which will include implementation of the program across a number of settings to assess the feasibility of the program and the initial impact of training on learners and patients/residents. Based on the feedback from study participants, the RCfT will include evaluation of patients’ experiences of compassion, the inclusion of a learner toolkit, engagement with key stakeholders prior to, during, and after the intervention, and the inclusion of each of the measures applied within the current study. In doing so, the EnACT program provides HCPs, leaders, educators, and organizations with an immersive, concise, interactive, evidence-based training program for improving compassion in personal practice and within the practice culture.

## Conclusion

In addition to further fortifying the importance of compassion in healthcare, this study highlights the potential for the EnACT compassion training program in cultivating HCP compassion and creating the conditions within the organizational culture where these learnings can be supported and sustained. This study addresses many of the known limitations of existing compassion training programs through its: evidence-informed, competency-based; clinically relevant approach; utilization of valid assessment tools to measure learner and patient outcomes; and focus on delivering and sustaining learning within and outside the classroom.

## Data Availability

The datasets generated and/or analyzed during the present study are available from the corresponding author on reasonable request. The SCQ-HCPCSA, and other versions of the SCQ, can be downloaded from www.compassionmeasure.com.
